# Systematic Deletion of Homeobox Genes in *Podospora anserina* Uncovers Their Roles in Shaping the Fruiting Body

**DOI:** 10.1371/journal.pone.0037488

**Published:** 2012-05-25

**Authors:** Evelyne Coppin, Véronique Berteaux-Lecellier, Frédérique Bidard, Sylvain Brun, Gwenaël Ruprich-Robert, Eric Espagne, Jinane Aït-Benkhali, Anne Goarin, Audrey Nesseir, Sara Planamente, Robert Debuchy, Philippe Silar

**Affiliations:** 1 Université Paris-Sud, Institut de Genetique et Microbiologie UMR8621, Orsay, France; 2 CNRS, Institut de Genetique et Microbiologie UMR8621, Orsay, France; 3 Université Paris Diderot, Sorbonne Paris Cité, UFR des Sciences du Vivant, Paris, France; 4 Université Paris Descartes, Sorbonne Paris Cité, Paris, France; New York State Health Department and University at Albany, United States of America

## Abstract

Higher fungi, which comprise ascomycetes and basidiomycetes, play major roles in the biosphere. Their evolutionary success may be due to the extended dikaryotic stage of their life cycle, which is the basis for their scientific name: the Dikarya. Dikaryosis is maintained by similar structures, the clamp in basidiomycetes and the crozier in ascomycetes. Homeodomain transcription factors are required for clamp formation in all basidiomycetes studied. We identified all the homeobox genes in the filamentous ascomycete fungus *Podospora anserina* and constructed deletion mutants for each of these genes and for a number of gene combinations. Croziers developed normally in these mutants, including those with up to six deleted homeogenes. However, some mutants had defects in maturation of the fruiting body, an effect that could be rescued by providing wild-type maternal hyphae. Analysis of mutants deficient in multiple homeogenes revealed interactions between the genes, suggesting that they operate as a complex network. Similar to their role in animals and plants, homeodomain transcription factors in ascomycetes are involved in shaping multicellular structures.

## Introduction

Although often inconspicuous, Eumycota fungi constitute one of the dominant life forms of our planet. They inhabit nearly all biotopes and their total biomass is huge [1]. During evolution, they have diversified to an estimated one million or more species [2] and have adopted many lifestyles including saprotrophy, mutualistic interactions with plants, algae and animals, and parasitic association with nearly all eukaryote groups. These different lifestyles confer on the Eumycota a range of important ecological roles such as recycling the carbon present in dead plant materials, providing food for small animals, ensuring the health of soil and controlling the spread of other organisms.

**Figure 1:**
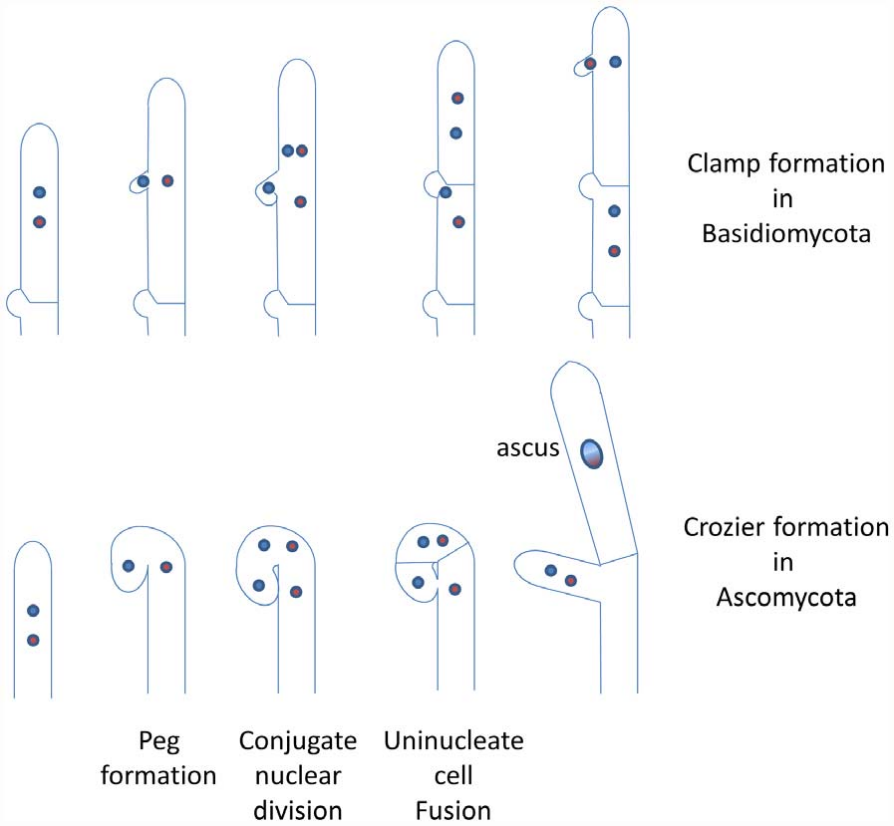
Development of clamps and croziers shares several features. In both cases, division starts with the formation of a peg, lateral in Basidiomycota and apical in Ascomycota. Conjugate nuclear division then follows, resulting in one cell with two nuclei and two cells with one nucleus. Uninucleate cells undergo anastomosis to recreate the dikaryotic conditions. In Basidiomycota, the apical cell continues its vegetative growth and undergoes further cell division. In contrast, in most Ascomycota, the apical cell usually differentiates into a meiocyte and undergoes karyogamy and meiosis, although in a few species it may continue to divide as a dikaryon.

Recent developments in our understanding of Eumycota phylogeny [3] have confirmed the old divide between “lower” (Chytridiomycetes and Zygomycetes in the old classification) and “higher” fungi (Basidiomycetes and Ascomycetes). The higher fungi, which encompass over 90% of the identified fungal species, form a monophyletic taxon, the Dikarya, whose name stems from the binucleate cell that forms during the lifecycle and undergoes mitotic division. In contrast to other eukaryotes, mating proceeds in two steps, plasmogamy and karyogamy, that are separated by numerous nuclear and cell divisions. Plasmogamy (the fusion of the two sexually competent haploid cells) gives rise to a cell with two nuclei: the dikaryon. The dikaryotic cell is able to divide while maintaining its dikaryotic condition with two sexually compatible nuclei. Eventually, the two haploid nuclei fuse in cells known as basidia and asci in the basidiomycetes and ascomycetes, respectively. Karyogamy is immediately followed by meiosis and spore formation. In most Dikarya, maintenance of the dikaryotic state relies on the ability of hyphae to fuse (anastomosis), a feature they share with the Glomeromycota [4]. Indeed, while some exceptions exist, mitosis in Dikarya usually gives rise to one binucleate and two uninucleate cells. The dikaryotic state is restored by fusion of the two uninucleate cells (Figure 1). The resulting structures are known as clamp connection in basidiomycetes and crozier in ascomycetes.

Although basidiomycetes and ascomycetes follow the same general pattern of dikaryophase maintenance, they also exhibit some differences. Foremost is the stage at which dikaryophase occurs. In most filamentous ascomycetes, the dikaryon forms following the fusion of male and female gametes and is located inside the fruiting body. Only in Taphrinomycotina, a basal sub-phylum of ascomycetes, have dikaryotic vegetative mycelia been described [5], [6]. In basidiomycetes, the dikaryon is formed by the fusion of two hyphae or two vegetative yeast cells, resulting in a mycelium in which all cells have two nuclei. This mycelium can grow for a very long time and eventually may differentiate fruiting bodies (such as mushrooms). Because of the similarities between clamp connection and crozier cytology (Figure 1), and the dissimilarities in the timing of dikaryophase and the fine details in the formation of clamps and croziers, it has been debated for over a century whether clamps and croziers are homologous or analogous. Deciphering the molecular mechanisms involved in clamp and crozier formation may help to resolve this longstanding issue.

Clamp connections are under the control of the mating-type in Basidiomycetes, which encodes either pheromones and receptors or homeoproteins (for a review see [7], [8]). The homeobox is a conserved DNA motif encoding a protein domain about 60 amino acids in length that promotes binding to DNA. Proteins containing this domain regulate transcription in a large array of eukaryotes (for a review see [9]). Homeoproteins have been identified in all major eukaryotic lineages, and the most probable evolutionary scenario is that they were present in ancestral eukaryotes with subsequent loss in some groups [10]. Homeodomain (HD) transcription factors have diversified into two superclasses, TALE (Three Amino acid Length Extension), which contain three extra amino acids between helix 1 and helix 2, and non-TALE. Interestingly, HD factors often function as homo- or hetero-dimers. In animals, they are present as dozens of paralogs, which include notably the hox genes, which participate in the definition of the body plan [11], [12]. Similarly, in plants, they are encoded by many paralogs [13] and participate in development programs [14].

Unlike the situation for basidiomycetes, there have been very few functional studies of HD proteins in filamentous ascomycetes (Pezizomycotina). The first HD protein characterized in filamentous ascomycetes was PAH1 from *Podospora anserina* [15]. Gene inactivation studies showed that PAH1 is involved in mycelium branching and the growth and production of male gametes. However, PAH1 has no roles in defining sexual identity or in sexual reproduction on the maternal side. In particular, ascospore production is normal in *pah1* mutants, suggesting that *pah1* does not control crozier formation. Deletion studies in *Neurospora crassa* [16] and *Magnaporthe grisea* [17] have also revealed roles for HD transcription factors in conidium and fruiting body development and in plant penetration. However, their role in crozier formation has not been assessed. Through targeted gene deletion, we show here that none of the seven HD transcription factors present in *P. anserina* are required for crozier formation, but that some are involved in shaping the fruiting body by acting in the maternal tissues. These results provide evidence for analogy and not homology between clamps of basidiomycetes and croziers of ascomycetes, and identify some of the factors controlling the shape of the fruiting bodies in ascomycetes.

## Results

### Identification of homeobox genes in *P. anserina*

**Figure 2:**
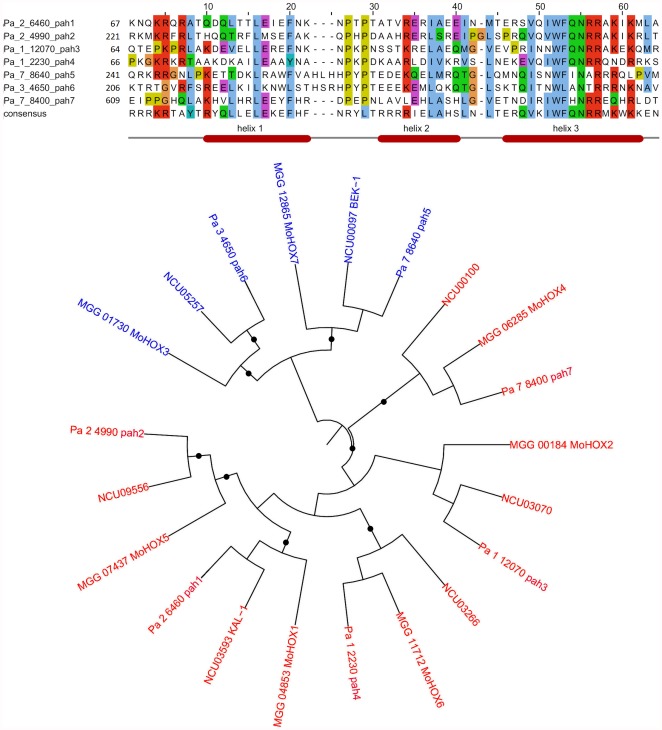
Evolution of HD transcription factors in Sordariomycetes. Top, alignment of the HD domains of the *P. anserina* HD transcription factors. The alignment was obtained with ClustalW2 [49] and colored according to the ClustalX color scheme provided by Jalview [50]. Numbers indicate the position in the protein of the first amino acids of the homeodomains. The bottom consensus line and the secondary structure are according to [9]. Bottom, PhyML tree of the HD factors of *P. anserina*, *N. crassa* and *M. grisea*. Black dots indicate statistically supported branches (>80%, 200 bootstraps). Non-TALE HD in red, TALE HD in blue.

A search of the *P. anserina* genome using the sequence of *pah1* and HD transcription factors from *N. crassa* and *M. grisea* [16], [17] as queries revealed the presence of seven genes coding for putative HD proteins. One of these genes (Pa_2_6460) corresponded to *pah1* (P. anserina homeobox 1) and six were novel genes (*pah2* to *pah7*; Figure 2). An additional protein with a homeo-related domain, which was homologous to the *S. cerevisiae* transcription factor Ste12p, was found. This protein, which has a highly divergent HD, was not considered further in this study as several lines of evidence suggest that Ste12p should not be considered as a homeobox protein [9].

Kim *et al.* [17] previously identified six of the *P. anserina* HD transcription factors and performed a phylogenetic analysis. Their data showed that PAH1 to PAH6 belong to six families that are likely to have diverged early in the evolution of the Pezizomycotina, although statistical support is too weak to ascertain whether these families are paralogous or orthologous to the other HD gene families. Each of the six genes was orthologous to *M. grisea* transcription factors. PAH7 was not identified by Kim et al. because the corresponding gene is located in a region that contained numerous sequencing errors. After correction of these errors, we were able to identify *pah7* as a seventh homeobox gene, which was orthologous to MoHOX4 ( = MGG_06285), the only *M. grisea* transcription factor without a *P. anserina* ortholog in the study by Kim et al. [17]. Our alignment (Figure 2) confirms that there are seven orthologous HD transcription factors in *M. grisea*, *N. crassa* and *P. anserina*, and shows that the HD proteins can be subdivided into two distinct classes, TALE and non-TALE [9]. PAH1 to PAH4 and PAH7 belong to the non-TALE class, while PAH5 and PAH6 belong to the TALE class (Figure 2). PAH7 has a non-canonical histidine at position 54, which usually contains glutamine, cysteine, serine or lysine in non-TALE proteins [9]. TALE-homeodomains are characterized by having three extra residues in the loop between helix 1 and helix 2, and by an isoleucine at position 54 [9]. PAH5 has a typical TALE homeodomain, while PAH6 contains an alanine at position 54 instead of the TALE-characteristic isoleucine. PAH6 also has a leucine instead of a phenylalanine at the highly conserved position 53 of the homeodomain (Figure 2). This striking difference is conserved in its orthologs in various Pezizomycotina (*e.g.*, *N. crassa*, *Sordaria macrospora*, *Chaetomium globosum*, *M. grisea*, *Aspergillus nidulans* and *A. fumigatus*). Transcriptomic analysis showed that all seven of the homeobox genes were expressed in *P. anserina* (Supporting Information S1).

### None of the HD transcription factors are involved in crozier formation

To investigate the role of the homeobox genes in *P. anserina*, we generated null mutants by targeted gene deletion for *pah2* to *pah7* (see Materials and Methods). *pah1* deletion mutants were already available [15]. Inactivation was performed in the Δ*mus-51* strain, which has an inactivated Ku70 subunit of the end joining complex [18] to optimize the targeting of each deletion cassette to its homologous locus. Successful deletion was confirmed by Southern blot hybridization (Supporting Information S1). The Δ*pah1 to* Δ*pah7* mutants were crossed to wild-type *P. anserina*. Purified deletion strains of both mating-types lacking the Δ*mus-51* marker were recovered in the progeny and selected for further analysis, thus ensuring that the observed phenotypes were not due to the inactivation of the *mus-51* gene.

**Figure 3:**
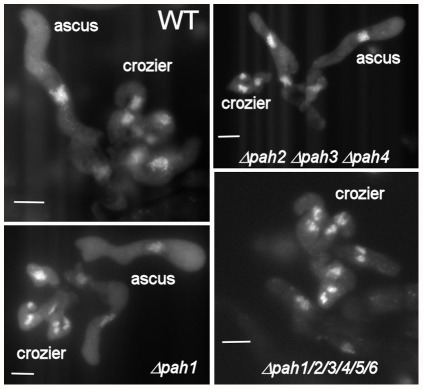
Crozier formation in the homeobox gene mutants. Perithecium centra were fixed, labeled with DAPI, and observed for the presence of croziers. Croziers were observed in all homeobox mutants, as shown here for Δ*pah1*. Some combinations of multiple mutants were also examined and found to differentiate croziers, as exemplified here by the Δ*pah2*Δ*pah3*Δ*pah4* triple mutant and the Δ*pah1*Δ*pah2*Δ*pah3*Δ*pah4*Δ*pah5*Δ*pah6* sextuple mutant.

To check whether HD transcription factors were involved in the maintenance of the dikaryotic stage, homozygous crosses for each homeobox gene deletion were set up. Ascospores could be recovered from all crosses, suggesting that all stages between fertilization and ascospore differentiation were normal when crossing homeobox gene mutants. To ensure that croziers were indeed formed during the sexual process, we made cytological observations of their formation (Figure 3). Although slight delays in their appearance occurred in some crosses, the croziers appeared normal in all cases, indicating that HD transcription factors are not essential for crozier differentiation. To address the potential issue of gene redundancy, we constructed all the double homeobox gene mutants (except for the *Δpah5 Δpah7* double mutants because the two genes are tightly linked), some triple mutants, and some other multiple mutants (construction is described in the Materials and Methods). Some of these mutants were sterile. However, as explained below, the sterility was due to a defect in the maternal tissue and not due to an inability to produce croziers and ascospores. Indeed, when the defect in the maternal tissue was complemented, croziers and ascospores were produced even in *Δpah1Δpah2Δpah3Δpah4Δpah5Δpah6* mutants (Figure 3).

### Phenotypic analysis of the *Δpah* deletion mutants

To determine the developmental stage at which homeobox genes are important, the seven deletion strains were submitted to a detailed phenotypic analysis during their vegetative growth and their sexual cycle. First, we determined whether the homeobox gene deletions had vegetative effects by examining mycelium growth, morphology and pigmentation on M2 minimal medium at 27°C (routine growth temperature). These experiments were also performed at 18°C and 35°C to test for possible cold- or thermo-sensitivity. The Δ*pah1* mutation was previously shown to affect hyphal extension and branching, leading to colonial cultures [15]. In contrast, deletions of the six other homeobox genes did not impair vegetative growth. Moreover, none of the mutants displayed cold- or thermo-sensitivity. Specific vegetative phenotypes well-established in *P. anserina* were examined next, including life span [19], ability to differentiate appressorium-like structures [20] and hyphal interference with *Penicillium chrysogenum* [21]. These assays did not reveal any differences between the deletion mutants and wild-type strains.

We then tested the efficiency of sexual reproduction in the mutant strains, including the formation of sexual cells and the differentiation of fertilized fruiting bodies. Although none of the HD transcription factors were involved in crozier formation, alterations in the sexual cycle were detected in some mutants. The homeogene deletion mutants were tested for impaired formation of spermatia, the *P. anserina* male gametes. The Δ*pah1* mutant is known to produce more spermatia than the wild-type strain [15]. Using the same assay as that used previously for testing the Δ*pah1* mutant [15], the other deletion mutants were found to have the same level of spermatia production as the wild-type strain. Moreover, the differentiation of female gametangia from ascogonia into protoperithecia was the same in the deletion mutants as in the wild-type. Hence, apart from an increased production of spermatia in the Δ*pah1* mutants, none of the mutations affected sexual events occurring before fertilization.

**Figure 4:**
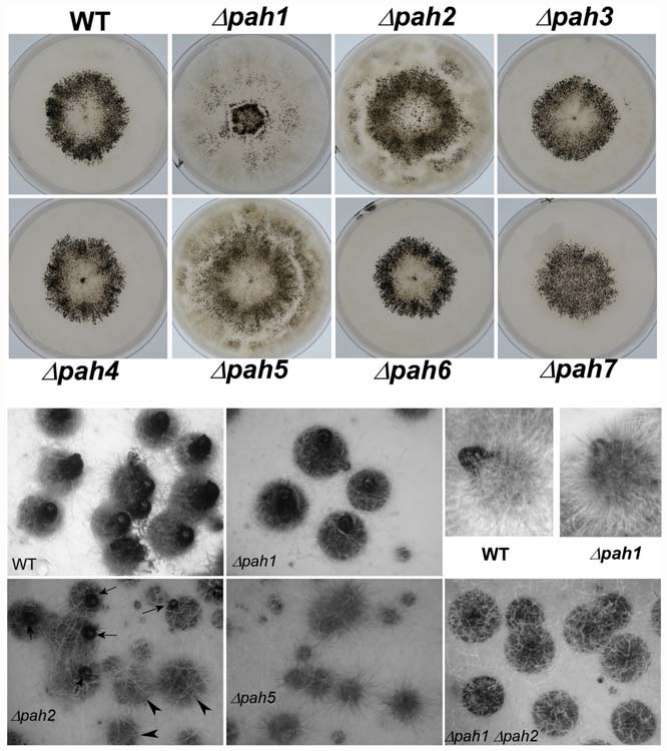
Fertility of the homeobox gene mutants. Repartition of fruiting bodies in homeobox mutants (top). Eight-centimeter M2 plates were inoculated in the center with mat+/mat− heterokaryotic cultures and incubated for seven days. Perithecia are visible as small black dots. Morphology of fruiting bodies in the Δ*pah* mutants (bottom). Images are from representative fruiting bodies from the plates in the top panel. For Δ*pah2*, arrows indicate perithecia with neck and arrowheads indicate perithecia without neck. WT, wild type.

The effects of the homeogene mutations on sexual development after fertilization were tested by examining *mat+*/*mat−* heterokaryons that were homozygous for a homeobox gene deletion. Wild-type *mat+/mat−* heterokaryotic culture inoculated in the center of an M2 plate differentiates perithecia mostly in a ring area 1 cm thick and located about 1 cm away from the inoculation point (Figure 4). A similar pattern was observed for the Δ*pah3*, Δ*pah4* and Δ*pah6* mutants, which differentiated fruiting bodies in the same way as the wild type (Figure 4). However, the pattern was different for the other four mutants (Figure 4). This was especially striking for the Δ*pah7* mutant, as this alteration of perithecium distribution was the only altered phenotype that we detected in this strain. Indeed, Δ*pah7* mutant perithecia developed with the same kinetics and morphology as wild-type, while Δ*pah1*, Δ*pah2* and Δ*pah5* had alterations in mycelium growth or in the shape and timing of the appearance of perithecia. Δ*pah1* perithecia were less hairy and more globular than the wild type, while the Δ*pah5* and Δ*pah2* mutants differentiated perithecia without beaks (also called necks, Figure 4).

Perithecia with and without beaks were observed on Δ*pah2* mycelia (Figure 4 and Table 1). Both types of perithecia contained ascospores, but only those with a beak were able to expel them. Beak formation in Δ*pah2* crosses was delayed compared to wild-type crosses. In WT×WT crosses, the development of fertilized protoperithecia into mature perithecia expelling ascospores took four days. The formation of the perithecial beak started 36 h post-fertilization and evolved so that the neck and the ostiole were well-formed 48 hours after fertilization. The shape of the perithecium changed at that time from globose to pyriform. In Δ*pah2*×Δ*pah2* crosses, this sequence was delayed by about 12–24 hours. Accordingly, the number of perithecia without a beak was reduced when incubation was extended beyond four days: from 51% after 4 days, to 32% after 5 days, and 29% after 7 days.

**Table 1:** Frequency of perithecia without neck in crosses homozygous for the Δ*pah* mutations.

Parental strain	% perithecia without neck^a^
*WT*	0
Δ*pah1*	0
Δ*pah2*	29±10*
Δ*pah3*	0
Δ*pah4*	0
Δ*pah5*	100
Δ*pah6*	0
Δ*pah7*	0
Δ*pah1* Δ*pah2*	96±5**
Δ*pah2* Δ*pah3*	44±5*
Δ*pah2* Δ*pah3* Δ*pah4*	44±5*
Δ*pah1* Δ*pah2* Δ*pah3*	88±10***
Δ*pah1* Δ*pah2* Δ*pah7*	96.5±5***
Δ*pah1* Δ*pah2* Δ*pah3*Δ*pah4*	90.2±5**

a Each cross was performed in duplicate on two separate Petri dishes inoculated with *mat+* and *mat−* strains. When the mycelia were confluent, sterile water was poured to disperse microconidia over the surface of the cultures and promote fertilization of female organs of the compatible mating-type. 5 to 9 independent counts were made seven days after fertilization on 150 to 400 perithecia formed on different sectors of the two dishes. Data from one*, two** or three*** assays were pooled. Δ*pah2* Δ*pah4*, Δ*pah2* Δ*pah6* and Δ*pah2* Δ*pah7* had the same % of perithecia without neck as Δ*pah2*. All the perithecia from Δ*pah1* Δ*pah5*, Δ*pah2* Δ*pah5*, Δ*pah3* Δ*pah5*, Δ*pah4* Δ*pah5* and Δ*pah6* Δ*pah5* had no neck. All the perithecia from the other double mutants had a beak.

No perithecium on Δ*pah5* mycelia had a beak (Figure 4 and Table 1), even after prolonged incubation, with the result that no ascus was expelled in crosses involving Δ*pah5* as the female parent. Perithecia nevertheless enlarged, although not as large as in the wild-type, and examination under the microscope showed that ascospores were formed, albeit with a 24 hour delay compared to the wild type.

**Figure 5:**
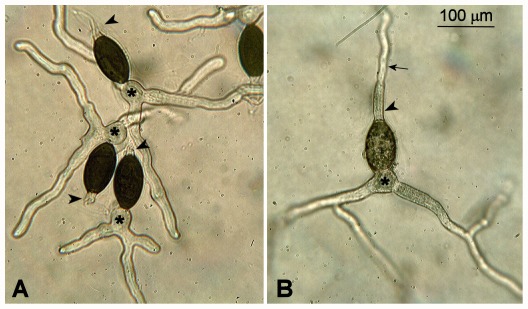
Germination of WT and Δ*pah5* ascospores. Ascospores were incubated for 6 h at 27°C on germination medium and observed by light microscopy. **A.** WT ascospores from a WT *mat+* × WT *mat−* cross are melanized and germ tubes originate from a germination peg (*) located at the pole opposite to the primary appendage (arrowhead). **B.** Δ*pah5* ascospores from a trikaryotic culture (see Results) are not fully melanized and germ tubes appear at both poles (arrow points at the germ tube originating from the primary appendage (*)). Numerous Δ*pah5* ascospores did not germinate even after incubation for several days.

In addition to the beak defect, there was also a defect in ascospore pigmentation. It was investigated in ♂ Δ*pah5*×♀ WT crosses, which had normal perithecia and expelled abundant progeny (Figure 5). Genetic analysis demonstrated that this phenotype was due to the Δ*pah5* mutation and that it was recessive (the heterokaryotic Δ*pah5/pah5^+^* ascospores, obtained during the second division segregation of the *pah5* locus in *P. anserina* asci, are black, as are the wild-type ascospores) and ascospore autonomous. The pigmentation defect had a variable expressivity, and Δ*pah5* ascospores could be white, light green or dark green.

The poorly pigmented Δ*pah5* ascospores also had a low germination rate. To quantify the germination rate, asci were recovered after their ejection from the perithecia on a layer of germination medium solidified by 1.2% agar. The layer was then observed under the light microscope after incubation for 6 h at 27°C. In these conditions, WT ascospores germinated with almost 100% efficiency, while the germination rate of Δ*pah5* ascospores was reduced to 42% (60/144 ascospores). Furthermore, Δ*pah5* ascospores had an altered mode of germination (Figure 5). While the germination of wild-type ascospores was unidirectional, with the germ tube originating at the pole opposite the primary appendage, the germination of Δ*pah5* ascospores was always bidirectional, with germ tubes originating from both tips of the spore.

To demonstrate that the phenotypes of the Δ*pah2* and Δ*pah5* mutants were actually due to the inactivation of the *pah2* and *pah5* genes, we introduced wild-type copies of these genes into the mutants (see Materials and Methods). In both cases, we observed a complete restoration of the wild-type phenotype. Therefore, while PAH1 appears important for vegetative growth and male gamete formation, PAH2 and PAH5 are important for the proper differentiation of perithecium necks and for the normal timing of sexual reproduction. Moreover, PAH5 is also required for proper ascospore maturation. PAH7 is involved solely in the patterning of perithecium production. The remaining three HD transcription factors, PAH3, PAH4 and PAH6, are dispensable under standard laboratory conditions.

### The defect in neck differentiation is due to a maternal defect

In Δ*pah2* and Δ*pah5* crosses with the wild-type strain, beakless perithecia were found only on mutant mycelia, whereas normal perithecia ejecting abundant progeny were observed on wild-type mycelia. Both mutants displayed a maternal defect because the perithecial walls and necks consisted of haploid maternal tissue. Similarly, in Δ*pah1*×wild-type crosses, globose perithecia were only found on the Δ*pah1* mycelium. Maternal effect was further examined by grafting wild-type perithecia onto Δ*pah1*, Δ*pah5* and Δ*pah2* mycelia. When wild-type perithecia were grafted onto wild-type mycelia, their development was similar to non-grafted perithecia, as previously described [22]. Similarly, when wild-type perithecia were grafted onto Δ*pah1*, Δ*pah5* and Δ*pah2* mycelia, their development was similar to perithecia grafted onto wild-type mycelia. Perithecia from Δ*pah1*×Δ*pah1*, Δ*pah2*×Δ*pah2* and Δ*pah5*×Δ*pah5* crosses grafted onto wild-type mycelia did not undergo wild-type development but resembled the non-grafted mutant perithecia: Δ*pah1*×Δ*pah1* perithecia remained less hairy and more globular, and neckless Δ*pah2*×Δ*pah2* and Δ*pah5*×Δ*pah5* perithecia did not acquire a neck. This indicated that the defect in each of these mutants could not be complemented by wild-type mycelium and, thus, that these transcription factors are likely to be autonomously expressed in the perithecial wall and beaks.

To test whether the Δ*pah5* beak defect could be rescued by wild-type maternal hyphae, a trikaryotic culture was generated by inoculating a Petri dish with a mixture of fragmented mycelia from the Δ*pah5 mat+*, Δ*pah5 mat-* and *pah5^+^*Δ*mat* strains. The latter is a mutant strain lacking the mating-type sequence [23]. Since the Δ*mat* nuclei carrying the functional *pah5* gene were unable to participate in fertilization, complementation was only possible in the maternal haploid tissues that form the wall and beak of the fruiting bodies. In addition to small perithecia without beaks, numerous large perithecia with beaks were observed in the trikaryotic cultures, showing that complementation occurred and that the PAH5 protein was required in the maternal perithecial tissues to trigger beak formation, thus confirming the conclusions from the grafting experiments.

### Phenotypic analysis of the double deletion mutants

Because HD transcription factors can form dimers, we tested for functional relationships between them by examining all double mutants (except Δ*pah5* Δ*pah7*, see Materials and Methods) during vegetative growth and the sexual phase. This analysis led to five main conclusions. First, no novel phenotype was found and thus no new function could be attributed to the HD proteins. Second, all double mutants containing Δ*pah1* had the same colonial phenotype as the Δ*pah1* single mutant. Third, the maternal sexual defect caused by Δ*pah5* was exacerbated in Δ*pah1*Δ*pah5*, Δ*pah2*Δ*pah5* and Δ*pah3*Δ*pah5* double mutants. Macroscopically, the double mutants produced perithecia that were smaller and darker than those produced by the Δ*pah5* single mutant. Furthermore, perithecia from the Δ*pah2*Δ*pah5* double mutant were barren: when they were squashed and observed under a light microscope, no ascogenous hyphae, asci or even paraphyses were detected. The trikaryotic Δ*pah2*Δ*pah5 mat+/*Δ*pah2*Δ*pah5 mat-/*Δ*mat* mycelia were fully fertile, indicating that the sterility was due to a defect in the maternal tissue and not the sexual tissue. Fourth, the impact of the Δ*pah2* mutation on the formation of perithecial beaks was modified when it was associated with Δ*pah3* or Δ*pah1* (Figure 5 and Table 1). In the Δ*pah2*×Δ*pah2* cross, the perithecia without beaks represented 29% of the perithecium seven days after fertilization. The frequency of perithecia without beaks was increased to 44% in the Δ*pah2*Δ*pah3* crosses and, more strikingly, was increased to 96% in the Δ*pah1*Δ*pah2* crosses. As observed for the Δ*pah5* neck defect, complementation with Δ*mat* hyphae restored normal neck production. Fifth, differential interactions between *pah7* and the other HD genes were detected for the production of a ring or a disk of perithecia. The Δ*pah3*Δ*pah7* mutants differentiated a disk of perithecia, the Δ*pah2*Δ*pah7* and Δ*pah6*Δ*pah7* mutants differentiated a ring, and the Δ*pah4*Δ*pah7* mutant had an intermediate phenotype.

Finally, when wood shavings were used instead of dextrin in the growth medium, even more pronounced interactions between deletion mutants were observed (Supporting Information S1). The Δ*pah5*Δ*pah1* and Δ*pah5*Δ*pah2* double mutants did not differentiate perithecia even after prolonged incubation, although mycelium growth did occur on this medium.

### Phenotypic analysis of the multiple deletion mutants

To further explore the genetic interactions between the homeobox gene mutations, we generated by genetic crosses three triple mutants, Δ*pah1*Δ*pah2*Δ*pah3*, Δ*pah1*Δ*pah2*Δ*pah7* and Δ*pah2*Δ*pah3*Δ*pah4*; the Δ*pah1*Δ*pah2*Δ*pah3*Δ*pah4* quadruple mutant; the Δ*pah1*Δ*pah2*Δ*pah3*Δ*pah4*Δ*pah5* quintuple mutant; and the Δ*pah1*Δ*pah2*Δ*pah3*Δ*pah4*Δ*pah5*Δ*pah6* sextuple mutant. Some interactions between the genes were observed when the percentages of perithecia without beaks were evaluated (Table 1). A slight but consistent decrease was seen in the percentage of perithecia without beaks in the Δ*pah1*Δ*pah2*Δ*pah3* triple mutant compared to the Δ*pah1*Δ*pah2* double mutant (88% instead of 96%). The Δ*pah1*Δ*pah2*Δ*pah7* triple mutant had a similar percentage (95.5%) to the Δ*pah1*Δ*pah2* double mutant. Thus, Δ*pah3* appeared to antagonize the cooperative effect of Δ*pah1* and Δ*pah2*, which contrasts with the synergistic interaction it displayed with Δ*pah2* in the Δ*pah2*Δ*pah3* double mutant. The percentage of perithecia without beaks was not further modified (90.5%) when a fourth mutation, Δ*pah4*, was added (Δ*pah1*Δ*pah2*Δ*pah3*Δ*pah4* quadruple mutant). Similarly, the addition of the Δ*pah4* mutation did not modify the Δ*pah2*Δ*pah3* interaction (44% perithecia without beaks in both the Δ*pah2*Δ*pah3*Δ*pah4* triple mutant and the Δ*pah2*Δ*pah3* double mutant).

The Δ*pah1*Δ*pah2*Δ*pah3*Δ*pah4*Δ*pah5* quintuple and the Δ*pah1*Δ*pah2*Δ*pah3*Δ*pah4*Δ*pah5*Δ*pah6* sextuple mutants differentiated only tiny fruiting bodies without ascospores, indicating that defects in the perithecial wall and beaks increased as more deletions were associated with the Δ*pah5* deletion. As for Δ*pah5*, this defect could be fully rescued by Δ*mat* hyphae, which enabled normal development of the fruiting body in the quintuple and sextuple mutants (Supporting Information S1).

## Discussion

This study is the second to account for the functions of the whole repertoire of HD proteins in a filamentous ascomycete fungus. However, in their study of *Magnaporthe grisea*, Kim *et al* [17] focused on the roles of these factors during asexual spore production and invasion of the host by this phytopathogen. In contrast, our study focused mostly on their role during sexual reproduction. Indeed, *P. anserina* disperses only by sexual reproduction and we therefore could not assess the role of HD proteins in asexual sporulation. It is noteworthy that none of the mutants had impaired production of spermatia, which closely resemble conidia but are unable to germinate. The Δ*pah1* mutant even had enhanced production of spermatia [15]. In contrast, MgHOX2, which is orthologous to PAH3, is necessary for conidium production in *M. grisea* [17]. These results suggest that spermatia and conidia may not be homologous structures. Although *P. anserina* lives as a saprobe on herbivore dung, it is also able to differentiate appressorium-like structures to penetrate plant materials [20]. None of the homeogenes are implicated in the production of these structures, while MgHOX7 (orthologous to PAH5) is required for appressorium formation in *M. grisea* [17]. However, PAH5 is involved in the development of ascospores, with specific roles in their melanization and germination. Homology between maturation of the *P. anserina* ascospore and differentiation of the *M. grisea* appressorium has been previously suggested [24], which is consistent with the results presented here. One feature found in both *P. anserina* and *M. grisea* is the slow growth, reduced aerial hyphae and the hyper-pigmented phenotypes of the deletion mutants for the orthologs PAH1 and MgHOX1. Slow growth and reduced aerial branching were also observed for mutants of the ortholog in *Neurospora crassa*, *kal-1* [16]. Slow growth in the *kal-1* mutant correlated with increased branching, as was also observed in the *P*. *anserina pah1* mutant, suggesting that the function of this transcription factor is conserved during Sordariomycetes evolution.

With regard to sexual development, we showed that deletions in four of the homeogenes could modify perithecium maturation, indicating a major role for these transcription factors in the development of multicellular structures, as previously described in animals and plants [12], [25]. The *pah1* deletion affected the shape of the fruiting body and the *pah7* deletion affected the area on which the fruiting bodies differentiate. Inactivation of *pah2* altered beak formation with an incomplete penetrance and the *pah5* deletion abolished beak formation and impaired growth of the fruiting body. For *pah5*, the same phenotype was previously reported for the deletion of the ortholog *bek-1* in *N. crassa* [16], arguing for a conservation of function of Bek-1/PAH5 during evolution of the Sordariales.

Necks are hallmarks of perithecia and differentiate them from other types of fruiting bodies in Ascomycetes. Apothecia are open fruiting bodies, while cleistothecia are completely enclosed ones. Both of these fruiting bodies lack necks. Intriguingly, while a functional *pah5* gene is present in the sequenced genomes of Eurotiomycetes, a class of Ascomycota that produces cleistothecia, no *pah2* appears to be present in the genomes of these fungi [17] (P. Silar, unpublished observation). It remains to be determined whether the lack of *pah2* is linked to the type of fruiting body differentiated by Eurotiomycetes and whether different homeobox genes have been recruited during evolution in the various classes of Ascomycota to shape the various types of fruiting bodies.

Our analyses of double and multiple mutants revealed interactions between the homeobox genes for neck differentiation and for production of a ring or disk by fruiting bodies. However, it appears impossible to propose a coherent model that positions the genes on a linear pathway or on intersecting pathways. For example, deletion of *pah3* increases the effect of the *pah2* deletion on necks but antagonizes the effect of the Δ*pah1*Δ*pah2* double deletion. Thus, the homeobox genes in *P. anserina* are more likely to form an interacting network than a linear pathway. Some of the interactions were greatly amplified on medium containing wood shavings as sole carbon source. Because the process of sexual development was longer on wood shavings medium, lack of fruiting bodies in the Δ*pah1*Δ*pah2* and Δ*pah2*Δ*pah5* double mutant could be due to an extended maturation time, yet even with prolonged incubation, we did not observe perithecia. It is possible that some of the HD transcription factors are involved in the response of the mycelium to the nutrient source, explaining why we did not observe clear phenotypes associated with the deletions of *pah3*, *pah4* and *pah6*. Different media and/or conditions might be required to see an effect of these gene deletions.

Since their discovery, there has been debate about whether clamps and croziers are analogous or homologous. Analogy is the opinion of, for example, Buller [26] and Schaffer [27]; the latter stated in his 1974 presidential address to the Mycological Society of America: “*I admit no homology between crozier and clamp connection, between ascospore and basidiospore, and between ascocarp and basidiocarp. The members of each of these pairs of structures seem to represent different, even if in part superficially similar, solutions to a single problem faced by both fungal groups*”. On the other hand, homology is supported by Martens [28] and others, and appears to be the prevailing opinion in present day textbooks. For example, Webster and Weber wrote on page 512 of their excellent textbook [29]: “*Clamp connections, characteristics of dikaryotic hyphae of basidiomycetes, are seen as homologous to the croziers in ascogenous hyphae, both have the same function of redistributing nuclei*”. Similarly, in the “Tree of Life” description of Basidiomycota (http://tolweb.org/Basidiomycota/20520/2007.04.20), it is written that “*Sexually reproducing Ascomycota also form dikaryons, although they are not as long-lived as those of Basidiomycota. The clade that includes Ascomycota and Basidiomycota has been called the Dikaryomycotina, reflecting this presumably homologous similarity… Croziers may be homologous to clamp connections*”.

Unlike basidiomycetes, for which the homeobox genes present at one of the mating-type loci are mandatory for clamp formation, homeodomain transcription factors appear dispensable for crozier formation in the filamentous ascomycete *P. anserina*. In basidiomycetes, the initial stages, including nuclear pairing, initial clamp cell formation, conjugate mitosis and first septum formation, are regulated by homeodomain transcription factors located at one of the two mating-type loci in tetrapolar Basidiomycetes (e.g., the A locus of *Coprinopsis cinerea*). The later stages of peg formation and cell fusion are controlled by the other mating-type locus (e.g., the B locus of *Coprinopsis cinerea*), which encodes pheromones and receptors. In the dimorphic pathogenic fungus *Ustilago maydis*, the formation of clamp-like structures is dependent on the homeodomain transcription factors encoded by the *b* mating-type locus, as in the higher basidiomycetes, while it is independent of the pheromone–receptor system encoded by the *a* mating-type locus [30]. Even deleting six HD genes in the same *P. anserina* strain had no impact on crozier formation. Ascomycetes homeobox genes may be orthologous or paralogous to those present in basidiomycetes. If the homeobox genes present in ascomycetes are paralogous to those of basidiomycetes, this means that key genes involved in the control of clamp formation are absent from ascomycetes. Alternatively, if ascomycetes homeobox genes are orthologous to those of basidiomycetes, then these genes are not involved in crozier formation. This result suggests that the molecular mechanisms controlling the formation of crozier in ascomycetes and clamp in basidiomycetes are different, suggesting that clamp and crozier are analogous rather than homologous structures. This has previously been proposed based on the many differences that exist in their formation, such as the fusion of the apex for crozier and of a lateral peg for clamp, and in the different stages of the lifecycle at which they take place [26].

**Figure 6:**
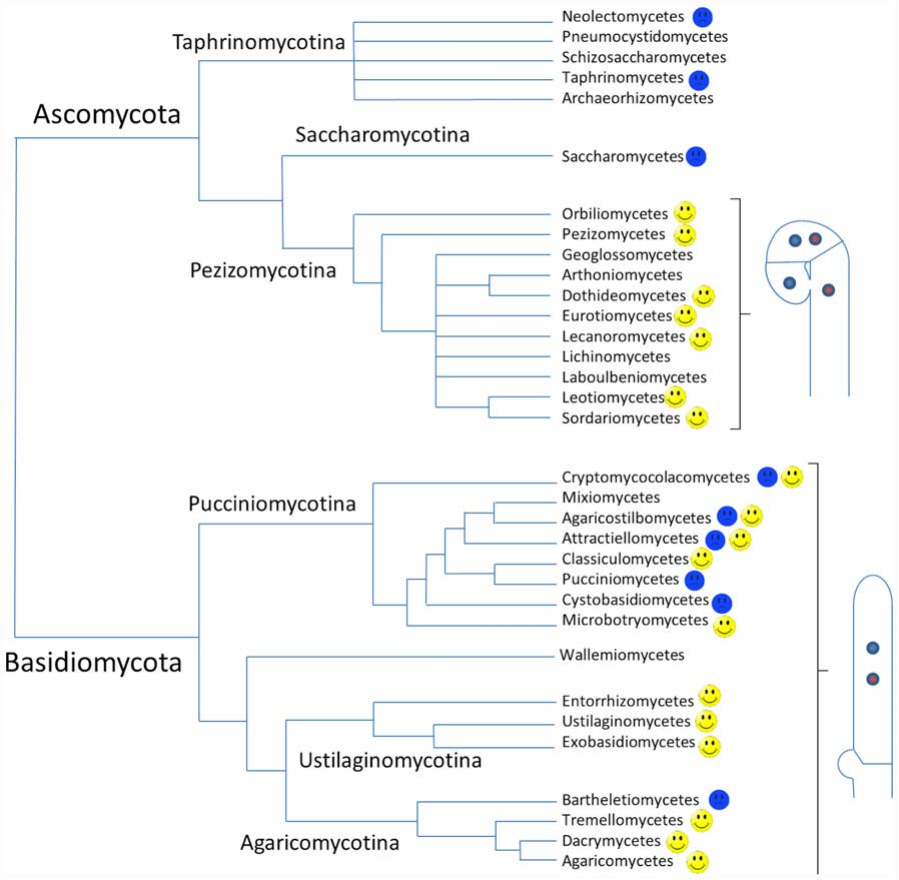
Phylogenetic tree of fungi and the presence of croziers and clamps. The tree is based on the latest phylogenetic data including those of James et al. [3]. In the Ascomycota, a smiling face indicates that croziers are present and a sad face indicates that they are absent. In the Basidiomycota, a smiling face indicates that clamps are present and a sad face indicates that they appear absent. No face is shown when we were not able to find data on the presence of croziers/clamps in the literature or that species from the group grow only as yeast.

Recent advances in understanding the phylogeny of fungi provide additional evidence for analogy (Figure 6). Among the three subphyla of Ascomycota, croziers are found only in the Pezizomycotina, which is thought to have evolved more recently. Croziers are not present in the Saccharomycotina and the Taphrinomycotina, although the latter contains a class of differentiating multicellular fruiting bodies, the Neolectomycetes [31]. While there are no reports of dikaryotic hyphae in Saccharomycotina, dikaryotic hyphae [5], [6], or hyphae containing paired nuclei [32], lacking clamp and crozier are found in the genus Taphrina. Therefore, the appearance of crozier in Pezizomycotina is more parsimonious than its independent disappearance in Saccharomycotina and Taphrinomycotina. The situation in Basidiomycetes appears more complex as the three subphyla contain groups with clamps and groups that lack them. Clamps are common in the Agaricomycotina and Ustilaginomycotina and much rarer in the Pucciniomycotina. This latter subphylum is thought to be basal to the other two [3]. For example, all Pucciniomycetes lack clamp [33], while *Cryptococolax abnorme*, another Pucciniomycotina belonging to the Cryptococolomycetes, has hyphae with clamp [34]. Yet, another Cryptococolomycetes, *Colacosiphon filiformis*, lack clamps [35]. Intriguingly, clamps are found only at the base of the probasidium in some species such as the Agaricostilbomycetes *Cystobasidiopsis nirenbergiae* [36] – a location resembling that of croziers. Therefore, clamps may have an ancient origin in the Basidiomycota, although the possibility that they appeared on more than one occasion during evolution cannot be excluded.

Although they play a major role in the biology of fungi, the presence and formation of clamp and crozier are neglected phylogenetic and molecular topics. Apart from the role of the mating-types, to our knowledge, only one gene, *clampless1*, has previously been identified as being involved in clamp formation based on studies in *Coprinopsis cinerea* [37], *Cryptococcus neoformans* [38] and *Ustilago maydis* [30], [39]. The *clampless1* gene is not conserved outside the basidiomycetes [30], [38]. The molecular determinants involved in crozier formation are less well known than those for clamp connection. The single gene identified as important for crozier formation, *cro1*, encodes a protein belonging to the family of UCS proteins, which are assumed to interact with various myosins [40]. The *cro1* mutants of *Podospora anserina* have a defect in the establishment and/or maintenance of a true cellular state: the actin belts and septa formation between the daughter nuclei after division are misplaced or absent, leading to a giant, multinucleated crozier [41]. Another gene possibly involved in crozier formation is *prm1*, which is involved in cell fusion [42]. Pheromones are not required for crozier formation in *P. anserina* [43]. This feature is unlike that of clamp connection in Agaricomycotina, suggesting again that crozier and clamp connection rely on different molecular pathways for their formation, further reinforcing the notion that clamps and croziers rely on different molecular mechanisms for their formation and hence are likely to be similar but independent solutions to the same problem, namely the faithful maintenance of the dikaryotic stage.

## Materials and Methods

### Strains and growth conditions

All strains of *P. anserina* used in this study were derived from the “S” (big S) wild-type strain, which is the strain used for sequencing of the *P. anserina* genome [44], [45]. The genome sequence and ESTs derived from the S strain are available at http://podospora.igmors.u-psud.fr. Details of the culture conditions and experimental procedures to work with this fungus can be accessed at the same internet address.

### Generation of deletion mutants

To delete the chromosomal copy of the homeobox genes (*pah2* to *pah5*), plasmids containing homeobox deletion cassettes conferring resistance to hygromycin B were constructed according to the *Asc*I/*Mlu*I method [46]. *Pah6* and *pah7* deletion cassettes were constructed according to the *N*. *crassa* strategy for high-throughput gene deletion [16], with modifications [46] (see Supporting Information S1 for primers). In the cases of *pah6* and *pah7*, a phleomycin resistance cassette was used. Each deletion plasmid was digested to generate the deletion cassette prior to transformation of Δ*mus-51* protoplasts [18]. Transformants were recovered on selective regeneration medium containing hygromycin B or phleomycin and subjected to PCR analysis to determine if the targeted gene was deleted. The PCR amplified the area of the junction specific to the replaced locus using two primers that anneal at an end of the selectable resistance gene (hygromycin B or phleomycin) and upstream of the proximal flank used in the deletion cassette (Supporting Information S1). Homologous recombination of the deletion cassette allows amplification of a predictable fragment on each side of the selectable resistance gene. Transformants with the putative correct deletion were obtained at high frequency in all transformation assays. Two or three candidate transformants were genetically purified by crossing them to wild-type *P. anserina*. This was done to eliminate potential untransformed nuclei and to segregate out the Δ*mus-51* mutation. Several *mat+* and *mat−* strains containing the deletion but lacking Δ*mus-51* were selected from each progeny and subjected to Southern blot analysis for validation (Supporting Information S1). *mat+* and *mat−* strains with the expected hybridization pattern were chosen as the stock deletion mutant for subsequent studies. To ensure that the phenotypes observed for Δ*pah2* and Δ*pah5* were actually due to inactivation of the *pah2* and *pah5* genes, wild-type alleles of these genes were reintroduced by transformation into the mutants. The *pah2* gene was obtained by amplification of a 4371 bp fragment encompassing exclusively the *pah2* gene (see primers in Supporting Information S1) and was used directly for co-transformation of the Δ*pah2* strain with the pPable vector [47]. The *pah5* gene was obtained by amplification of a 2314 bp fragment encompassing the *pah5* gene and the 3′ end of an adjacent gene (see primers in Supporting Information S1), followed by cloning of this fragment in the pPable vector [47].

### Generation of strains with multiple deletions

Double mutants were constructed by crossing single mutants of opposite mating-type in all pairwise combinations. In crosses involving *Δpah1* to *Δpah5* and either Δ*pah6* or Δ*pah7*, the double mutant progeny were resistant to both hygromycin B and phleomycin. For the other crosses, double mutants could not be identified based on a particular pattern of resistance. To identify these double mutants, we searched for asci exhibiting first division segregation (FDS) of the hygromycin phenotype, i.e., asci with two HygR (resp. PhleR) and two HygS (resp. PhleS) ascospores. Sensitive ascospore-derived cultures from these asci carried the wild-type alleles of both genes; hence, resistant cultures had mutations in both genes. A heterokaryotic *mat+*/*mat−* resistant culture was self-crossed and *mat+* and *mat−* homokaryotic double mutants were isolated from the progeny. Colonial growth (for Δ*pah1)* and ascospore pigmentation defect (for Δ*pah5)* constituted two easily scorable phenotypes, allowing easy identification of the corresponding mutations. In all crosses involving Δ*pah5*, it was used as the male partner since it exhibited a maternal sexual defect. To generate double mutants for *pah1* and *pah2*, which are closely linked on chromosome 2, 70 asci were analyzed and double mutants were isolated from tetratype asci. Only Δ*pah5*Δ*pah7* double mutants could not be recovered, which was due to the tight linkage (d<0.1 cM) of these two genes. The genotype of all double mutants was confirmed by PCR assays directed at the specific junctions for each deletion.

Triple mutants were constructed by crossing single and double mutants. Δ*pah2*Δ*pah3*Δ*pah4* was isolated from the Δ*pah2*×Δ*pah3*Δ*pah4* cross by screening asci exhibiting the 2 hygR/2 hygS segregation described above. Δ*pah1*Δ*pah2*Δ*pah3* mutants were isolated from Δ*pah1*Δ*pah2*×Δ*pah3* using a similar strategy. The Δ*pah1*Δ*pah2*Δ*pah3*Δ*pah4* quadruple mutants were isolated from Δ*pah1*Δ*pah2*×Δ*pah3*Δ*pah4*. Sixty-four spored asci were analyzed to determine their germination phenotype. Eight asci with 2 colonial/2 non-colonial segregation, indicative of Δ*pah1* FDS, were further tested on hygromycin B medium. One ascus with 2 hygR colonial/2 hygS non-colonial spores was identified. The *mat+* or *mat−* homokaryotic quadruple mutants were obtained in the progeny of one hygR colonial ascospore from this ascus. Presence of the four deletions was confirmed by PCR. The Δ*pah1*Δ*pah2*Δ*pah3*Δ*pah4*Δ*pah5* quintuple mutants were isolated from Δ*pah1*Δ*pah2*Δ*pah3*Δ*pah4*×Δ*pah5*. More than 100 asci with 2 pigmented/2 unpigmented ascopores (2*pah5^+^*/2Δ*pah5*) were analyzed on hygromycin B medium. Two asci with 2 hygR colonial/2 hygS non-colonial segregation pattern were identified. The quintuple mutant was generated from a hygR colonial culture from one of these two asci. The presence of all five deletions was confirmed by PCR analysis. The sextuple mutant Δ*pah1*Δ*pah2*Δ*pah3*Δ*pah4*Δ*pah5*Δ*pah6* was generated by crossing Δ*pah6* as female with the quintuple mutant as male. One hundred and sixty asci with 2 pigmented/2 unpigmented ascospores (2*pah5^+^*/2Δ*pah5*) were analyzed. Unpigmented ascospores gave rise to colonial mycelium (Δ*pah5*Δ*pah1*) in 18 asci, among which one ascus exhibited 2 hygR phleR/2 hygS phleR segregation. The hygR phleR ascospores were thus homokaryotic for Δ*pah1*Δ*pah2*Δ*pah3*Δ*pah4*Δ*pah5* and heterokaryotic for Δ*pah6. mat+* and *mat−* homokaryotic sextuple mutants were then isolated from the progeny of self-fertilization of one of the mycelia recovered from the hygR phleR ascospores. When Δ*pah5* was homozygous in a cross, the maturation defect of the perithecia was rescued with the Δ*mat* helper strain (see Results).

### Fertility analysis

The procedure for heterozygous crosses was as follows: mutant and wild-type strains of opposite mating-types were cultivated on opposite sides of the same Petri dish. After three days, when the cultures had invaded the whole dish, sterile water was poured onto the mycelia and gently dispersed over the surface of the cultures. As each *P*. *anserina* strain produces spermatia and protoperithecia whatever its mating-type, this procedure enables reciprocal fertilization of mutant and wild-type strains. Homozygous crosses of each mutant were performed either by inoculating *mat+* and *mat*- strains on opposite sides of the plate, as described above, or by inoculating a drop of mixed *mat+* and *mat−* mycelia at the center of the plate.

### Cytology

Cells were fixed in 7.4% paraformaldehyde and processed for fluorescence microscopy as previously described [48]. Mitochondrial DNA and nuclei were stained with DAPI (0.5 mg ml^−1^). Cells were observed in a three-dimensional deconvolution microscope (DM IRE2; Leica) equipped with an HCxPL APO 100× oil CS objective, NA = 1.40 (Leica). The images were captured by a 10 MHz Cool SNAPHQ charge-coupled device camera (Roper Instruments), with a z-optical spacing of 0.5 µm. METAMORPH software (Universal Imaging Corp.) was used to acquire z-series. Images were processed using ImageJ software (NIH, Bethesda).

## Supporting Information

Supporting Information S1 Figure S1. Expression of homeobox genes during fruiting body maturation. Differential expression in space and time of the seven homeobox genes was assessed by microarray analysis in a time course of mycelium growth (Bidard and Silar, unpublished data) and perithecium maturation (Bidard and Berteaux-Lecellier, unpublished data). No difference in expression was detected for the seven genes in one-day, two-day or three-day old mycelia. In contrast, statistically significant differential expression of the homeobox genes was detected during perithecium maturation. Each gene had its own pattern of expression. However, the expression of each homeobox gene except *pah7* increased after fertilization, which corresponded to the timing at which some of these genes are required for proper development of the fruiting bodies, i.e., for neck differentiation. The expression of most of the genes was decreased at the end of the development period (96 h). The expression level was measured as described by Bidard et al. [51] on three independent samples and was normalized by the median intensity for each gene. Figure S2. Southern blot analyses of the Δ*pah* mutants. DNA was isolated from wild-type *P. anserina* and one or several purified transformants (T1, T2, T3…) and digested with appropriate restriction enzymes. The blots were probed either with a sequence containing the relevant CDS and its flanking region (Δ*pah2*, Δ*pah3*, Δ*pah4*, Δ*pah5*) or with the *ble* sequence (Δ*pah6*, Δ*pah7*). For each *pah* gene, a restriction map of the wild-type and mutant locus is presented. The sizes of the expected fragments are indicated on the maps and are reported close to the corresponding fragment on the Southern blot. The DNA sequence around *pah7* is not well defined because of sequencing errors and the restriction pattern cannot be confidently predicted for the *pah7* deletion. To confirm the deletion of *pah7*, the blot was stripped and further probed with the *pah7* CDS to confirm that it was absent in the Δ*pah7* candidates. Figure S3. Fertility of Δ*pahx* mutants on medium containing wood shavings. Strains of the indicated genotypes were inoculated on medium containing wood shavings as the sole carbon source. The images were taken 10 days later. Even after prolonged incubation for up to three weeks, no perithecia were observed in the *Δpah1Δpah2* and *Δpah2Δpah5* cultures. The small dots visible on the *Δpah2Δpah5* cultures correspond to dark, insoluble product(s) excreted by the hyphae. These products are also excreted by wild-type and all the other *pahx* mutants. Figure S4. Fertility rescue by Δ*mat* hyphae. Heterokaryons with the following components: *pah1*Δ*pah2*Δ*pah3*Δ*pah4*Δ*pah5*Δ*pah6 mat+*, *pah1*Δ*pah2*Δ*pah3*Δ*pah4*Δ*pah5*Δ*pah6 mat−* and*Δmat* are able to differentiate fruiting bodies of wild-type appearance, which produce abundant ascospores.
(DOC)
