# Supplementary material for: Systematic Deletion of Homeobox Genes in Podospora anserina Uncovers Their Roles in Shaping the Fruiting Body
Source: PLoS One. 2012 May 25;7(5):e37488. doi: 10.1371/journal.pone.0037488 (PMC3360767; doi:10.1371/journal.pone.0037488)
Supplement: Supporting Information S1 — Figure S1. Expression of homeobox genes during fruiting body maturation. Differential expression in space and time of the seven homeobox genes was assessed by microarray analysis in a time course of mycelium growth (Bidard and Silar, unpublished data) and perithecium maturation (Bidard and Berteaux-Lecellier, unpublished data). No difference in expression was detected for the seven genes in one-day, two-day or three-day old mycelia. In contrast, statistically significant differential expression of the homeobox genes was detected during perithecium maturation. Each gene had its own pattern of expression. However, the expression of each homeobox gene except pah7 increased after fertilization, which corresponded to the timing at which some of these genes are required for proper development of the fruiting bodies, i.e., for neck differentiation. The expression of most of the genes was decreased at the end of the development period (96 h). The expression level was measured as described by Bidard et al. [51] on three independent samples and was normalized by the median intensity for each gene. Figure S2. Southern blot analyses of the Δpah mutants. DNA was isolated from wild-type P. anserina and one or several purified transformants (T1, T2, T3…) and digested with appropriate restriction enzymes. The blots were probed either with a sequence containing the relevant CDS and its flanking region (Δpah2, Δpah3, Δpah4, Δpah5) or with the ble sequence (Δpah6, Δpah7). For each pah gene, a restriction map of the wild-type and mutant locus is presented. The sizes of the expected fragments are indicated on the maps and are reported close to the corresponding fragment on the Southern blot. The DNA sequence around pah7 is not well defined because of sequencing errors and the restriction pattern cannot be confidently predicted for the pah7 deletion. To confirm the deletion of pah7, the blot was stripped and further probed with the pah7 CDS to confirm that it was absent in the Δpah7 cand [file pone.0037488.s001.doc]

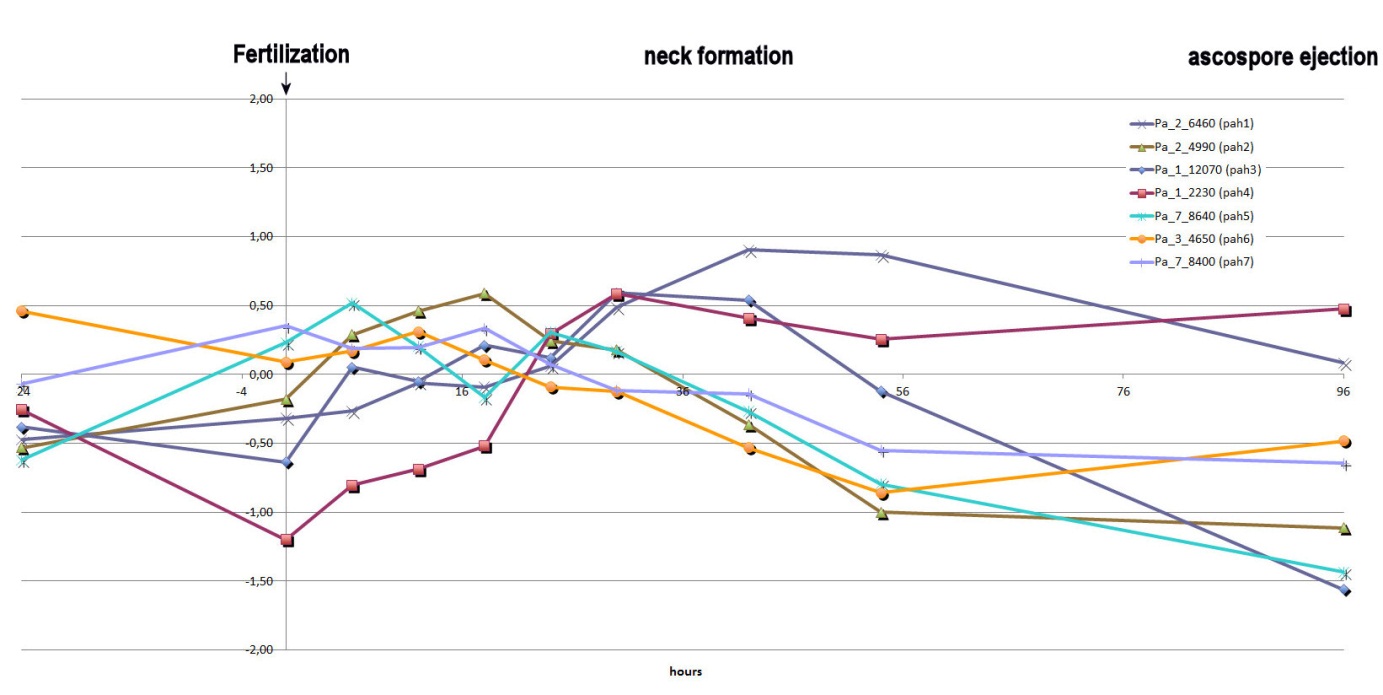


**Figure S1. Expression of homeobox genes during fruiting body maturation.** Differential expression in space and time of the seven homeobox genes was assessed by microarray analysis in a time course of mycelium growth (Bidard and Silar, unpublished data) and perithecium maturation (Bidard and Berteaux-Lecellier, unpublished data). No difference in expression was detected for the seven genes in one-day, two-day or three-day old mycelia. In contrast, statistically significant differential expression of the homeobox genes was detected during perithecium maturation. Each gene had its own pattern of expression. However, the expression of each homeobox gene except *pah7* increased after fertilization, which corresponded to the timing at which some of these genes are required for proper development of the fruiting bodies, i.e., for neck differentiation. The expression of most of the genes was decreased at the end of the development period (96 h). The expression level was measured as described by Bidard et al. on three independent samples and was normalized by the median intensity for each gene.


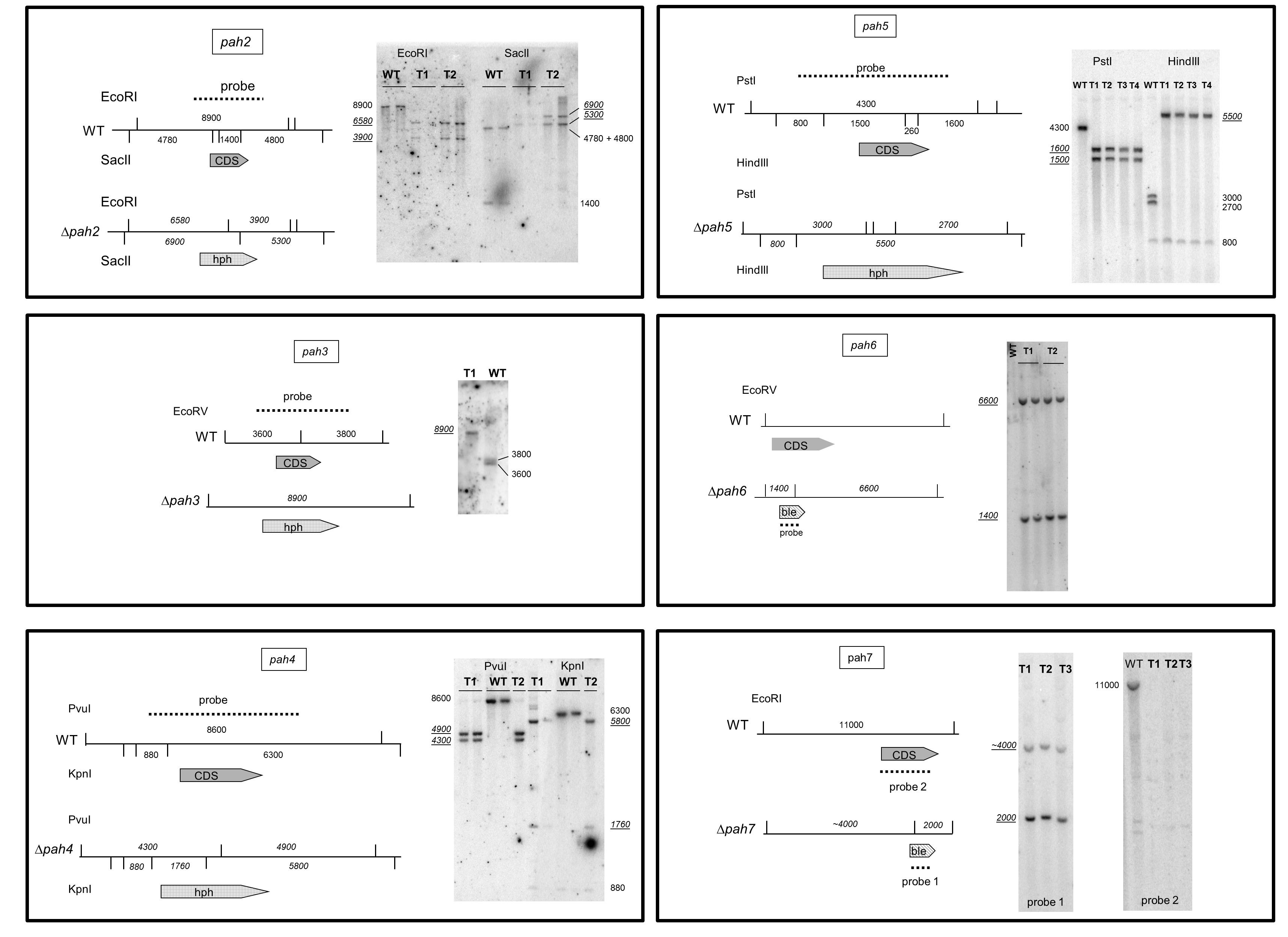


**Figure S2. Southern blot analyses of the Δ*pah* mutants.** DNA was isolated from wild-type *P. anserina* and one or several purified transformants (T1, T2, T3…) and digested with appropriate restriction enzymes. The blots were probed either with a sequence containing the relevant CDS and its flanking region (Δ*pah2*, Δ*pah3*, Δ*pah4*, Δ*pah5*) or with the *ble* sequence (Δ*pah6,* Δ*pah7*). For each *pah* gene, a restriction map of the wild-type and mutant locus is presented. The sizes of the expected fragments are indicated on the maps and are reported close to the corresponding fragment on the Southern blot. The DNA sequence around *pah7* is not well defined because of sequencing errors and the restriction pattern cannot be confidently predicted for the *pah7* deletion. To confirm the deletion of *pah7*, the blot was stripped and further probed with the *pah7* CDS to confirm that it was absent in the Δ*pah7* candidates.

**
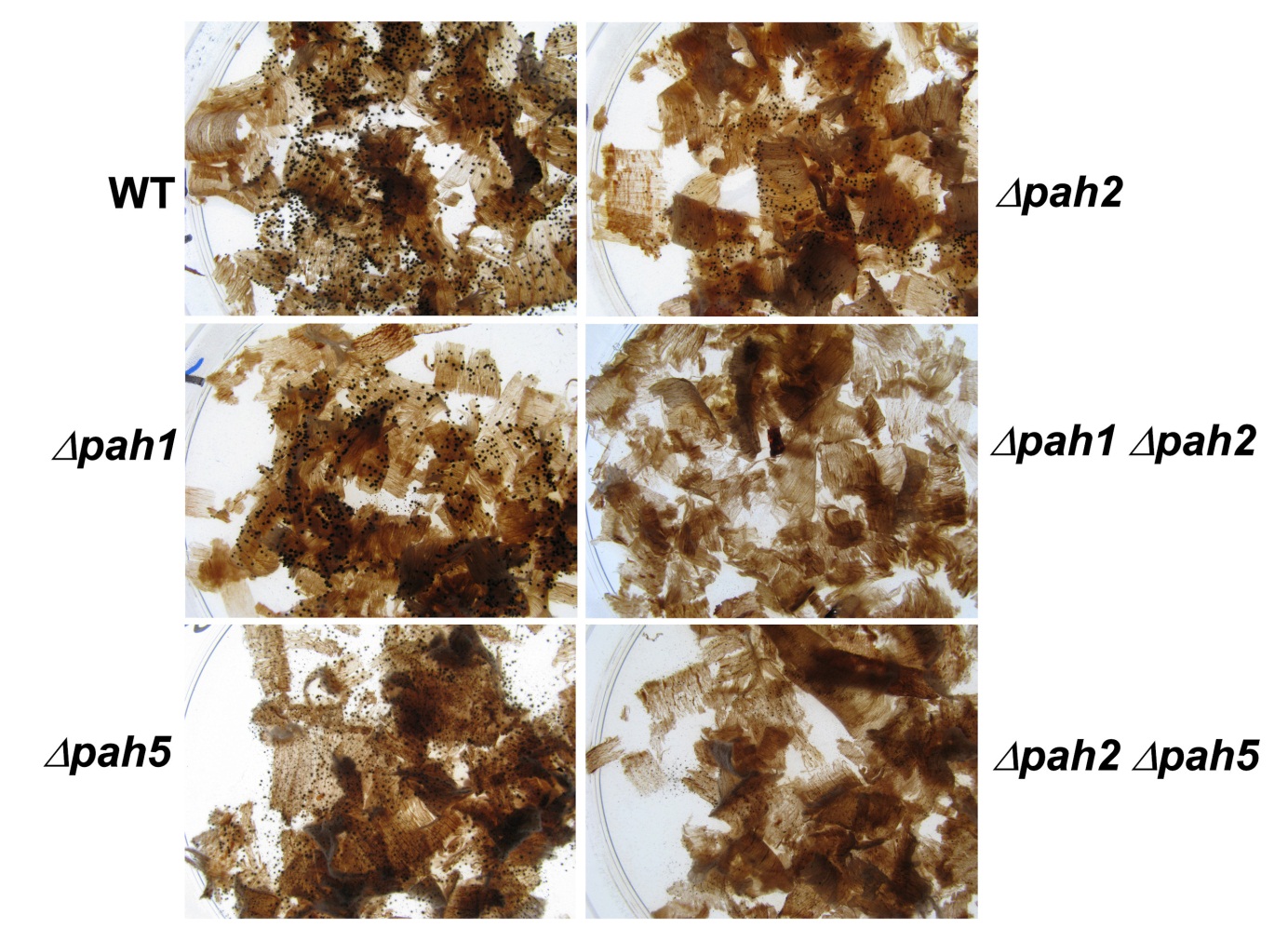
**

**Figure S3. Fertility of Δ*pahx* mutants on medium containing wood shavings.** Strains of the indicated genotypes were inoculated on medium containing wood shavings as the sole carbon source. The images were taken 10 days later. Even after prolonged incubation for up to three weeks, no perithecia were observed in the *Δpah1Δpah2* and *Δpah2Δpah5* cultures. The small dots visible on the *Δpah2Δpah5* cultures correspond to dark, insoluble product(s) excreted by the hyphae. These products are also excreted by wild-type and all the other *pahx* mutants.


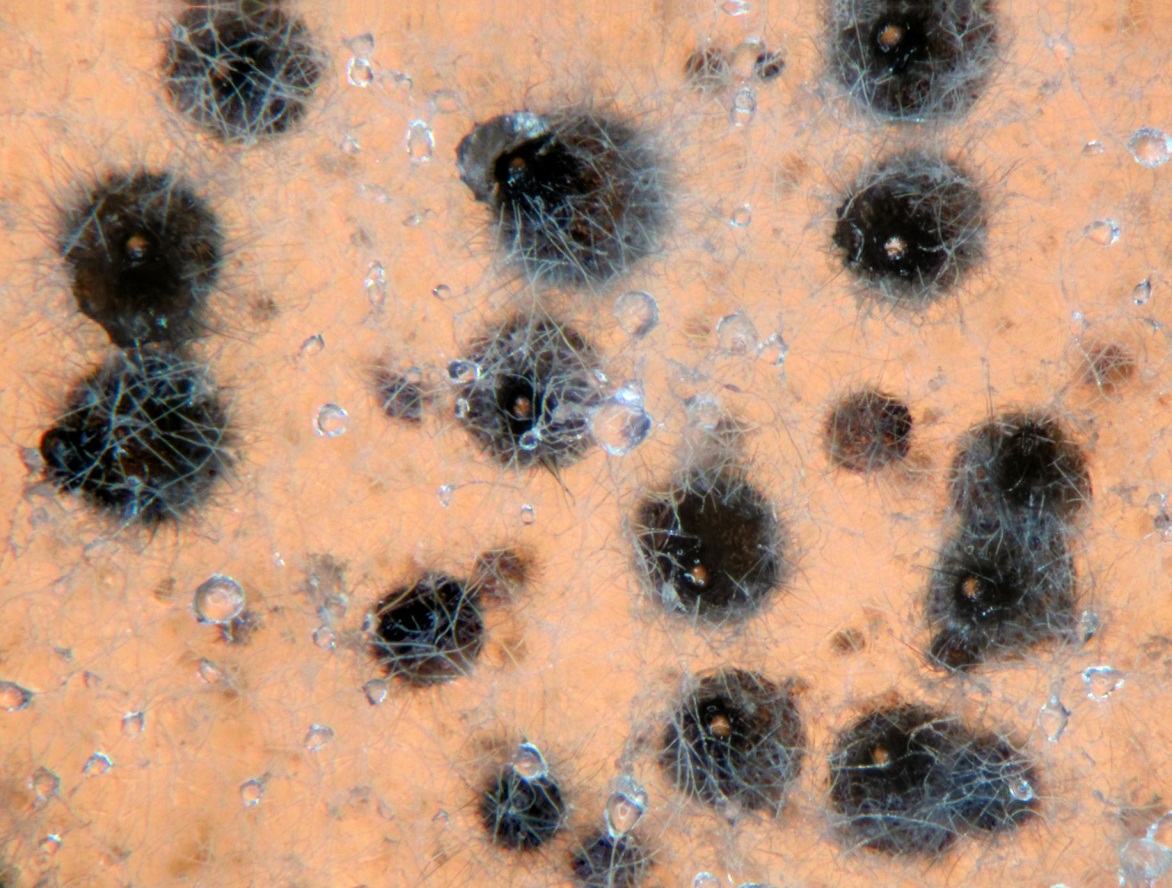


**Figure S4. Fertility rescue by Δ*mat* hyphae.** Heterokaryons with the following components: *pah1*Δ*pah2*Δ*pah3*Δ*pah4*Δ*pah5*Δ*pah6 mat+*, *pah1*Δ*pah2*Δ*pah3*Δ*pah4*Δ*pah5*Δ*pah6 mat-* and* *Δ*mat* are able to differentiate fruiting bodies of wild-type appearance, which produce abundant ascospores.

**Table S1**

| **Primer name** | **sequence^a^** | **use** |
| --- | --- | --- |
| 5_HOM2_Asc | aggcgcgccgggatggattgtcgaccgac | Deletion of *pah2* (Pa_2_4990) |
| 3_HOM2_Asc | tggcgcgccgagagcatcattttgggctac | Deletion of *pah2* (Pa_2_4990) |
| 5_HOM3_Asc | ggcgcgccatgatgtccaactgggtcgac | Deletion of *pah3* (Pa_1_12070) |
| 3_HOM3_Asc | ggcgcgccgtcagtgttctgattctgg | Deletion of *pah3* (Pa_1_12070) |
| 3_HOM4_Asc | ggcgcgccttggcttgctccatcaccttc | Deletion of *pah4* (Pa_1_2230) |
| 5_HOM4_Sma | ctgcccggggaggtgctgttgttctg | Deletion of *pah4* (Pa_1_2230) |
| 3_HOM4_Xba | tttctagactttctggagttggtg | Deletion of *pah4* (Pa_1_2230) |
| 5_HOM4_Asc | ggcgcgccgtaaaggagcttgg | Deletion of *pah4* (Pa_1_2230) |
| 5AscBEK | ggcgcgcctggttctcttgttaactgacc | Deletion of *pah5* (Pa_7_8640) |
| 5BEKSpe1 | ggactagtcccattcgatctcaacctgc | Deletion of *pah5* (Pa_7_8640) |
| 3AscBEK | ggcgcgccgaacacaacgtcaacgg | Deletion of *pah5* (Pa_7_8640) |
| 3ApaBEK | atgggcccggcaggtgattgcttgcctgg | Deletion of *pah5* (Pa_7_8640) |
| 4650_5f | gtaacgccagggttttcccagtcacgacggcgcgccggagagtctataacatcgc | Deletion of *pah6* (Pa_3_4650) |
| 4650_5r | gtggaagggaagggatgctcgaagaattccggagaaggacaggaaagg | Deletion of *pah6* (Pa_3_4650) |
| 4650_3f | gacgctcgaaggctttaatttgcaagcttgtgtatgatgcatggaaatctcc | Deletion of *pah6* (Pa_3_4650) |
| 4650_3r | gcggataacaatttcacacaggaaacagcggcgcgccatgacttctctctcttttg | Deletion of *pah6* (Pa_3_4650) |
| 5f_8400 | gtggaagggaagggatgctcgaagaattcggagaagctgactattgtc | Deletion of *pah7* (Pa_7_8400) |
| 5r_8400 | gtaacgccagggttttcccagtcacgacggcgcgccgagggtgtgagagattgtatc | Deletion of *pah7* (Pa_7_8400) |
| 3f_8400 | gacgctcgaaggctttaatttgcaagcttggacggggaataagtagatg | Deletion of *pah7* (Pa_7_8400) |
| 3r_8400 | gcggataacaatttcacacaggaaacagcggcgcgccgagcgttgggatgaattc | Deletion of *pah7* (Pa_7_8400) |
| 5_cpc1_Mlu | gcgacgcgtccgagatgcgccgcgtgc | Amplification of *hph* for *pah2, 3, 4, 5* deletion |
| 3_TtrpC | gctgtatctggaagaggtaaac | Amplification of *hph* for *pah2* deletion |
| 3_TtrpC_Mlu | gtcacgcgtagaggatcctctagcta | Amplification of *hph* for *pah3, 4, 5* deletion |
| blef | gaattcttcgagcatcccttc | Amplification of *ble* for *pah6* and *pah7* deletion. |
| bler | aagcttgcaaattaaagccttcgagcgtcc | Amplification of *ble* for *pah6* and *pah7* deletion. |
| 5_AM_cpc1 | gcacgcggcgcatctcggacgcgcc | Detection of the deletion of *pah2, 3, 4, 5.* |
| 3_MA_TtrpC | gctccatgtcaacaacgcgcc | Detection of the deletion of *pah2* |
| 3_T_trp | gtaatccttctttctagctagagg | Detection of the deletion of *pah3, 4, 5.* |
| HOM2-S0 | gttccatcaacttcctggcac | Detection of the deletion of *pah2* |
| HOM2-A0 | gcgtctagatctgaataagtgg | Detection of the deletion of *pah2* |
| 5 HOM3 | gcacgcagcagagcacaggttc | Detection of the deletion of *pah3* |
| 3 HOM3 | ggccaaactgttgtttctttgttc | Detection of the deletion of *pah3* |
| 5_HOM4 | gaccggataagaagtagccaag | Detection of the deletion of *pah4* |
| 3_HOM4 | ggagtggaccgctgaagatatc | Detection of the deletion of *pah4* |
| 3BEK | ggtaggaaggcgattagtggtttg | Detection of the deletion of *pah5* |
| 5BEK | gcaccagctgcttgtcctggttc | Detection of the deletion of *pah5* |
|  |  | Detection of the deletion of *pah6* |
|  |  | Detection of the deletion of *pah6* |
|  |  | Detection of the deletion of *pah7* |
|  |  | Detection of the deletion of *pah7* |
| 5_PAH2 | ctgccacgatagagttccg | Amplification of *pah2* for complementation. |
| 3_PAH2 | aggtgccctccatcttgtt | Amplification of *pah2* for complementation. |
| Eco_5_Beck | ggaattctggctggtgtcgatgtcatc | Amplification of *pah5* for complementation. |
| Bam_3_Beck | cgggatccagaccggtccaaaagg | Amplification of *pah5* for complementation. |

^a^: red letters: nucleotides corresponding to the pRS426 vector ; green letters: *Asc*I site; blue letters: nucleotides corresponding to the selectable phleomycin marker of pPable .

Supporting References

1. Bidard F, Imbeaud S, Reymond N, Lespinet O, Silar P, et al. (2010) A general framework for optimization of probes for gene expression microarray and its application to the fungus Podospora anserina. BMC Res Notes 3: 171.

2. Sikorski RS, Hieter P (1989) A system of shuttle vectors and yeast host strains designed for efficient manipulation of DNA in Saccharomyces cerevisiae. Genetics 122: 19-27.

3. Coppin E, Debuchy R (2000) Co-expression of the mating-type genes involved in internuclear recognition is lethal in *Podospora anserina*. Genetics 155: 657-669.
